# Function of *Brassica napus BnABI3* in *Arabidopsis gs1*, an Allele of *AtABI3*, in Seed Development and Stress Response

**DOI:** 10.3389/fpls.2019.00067

**Published:** 2019-02-05

**Authors:** Peipei Xu, Weiming Cai

**Affiliations:** Laboratory of Photosynthesis and Environment, CAS Centre for Excellence in Molecular Plant Sciences, Shanghai Institute of Plant Physiology and Ecology, Chinese Academy of Sciences, Shanghai, China

**Keywords:** *Brassica napu*s, functional complementation, freezing tolerance, desiccation tolerance, ER stress, root growth

## Abstract

Abscisic acid (ABA) has been implicated in plant adaptation to various environmental stresses in addition to the regulation of seed dormancy and leaf senescence. ABI3 is a B3 domain-containing family protein and functions in the ABA signaling pathway during seed development. To date, the ABI3 orthologous have not been studied in *Brassica napus*. The aim of this study is to investigate the function of *BnABI3* in plant development and stress response. Here, we identified an *Arabidopsis* line (*gs1*) from a population of mutagenized seeds and showed that GS1 is a new allele of *AtABI3*. When the *Arabidopsis gs1* mutant was transformed with the *BnABI3* gene, the transformed plants produced seeds that turned yellow and acquired desiccation tolerance. Moreover, *BnABI3* regulates seed coat development and mucilage secretion by directly targeting the *AtMUM1* and *AtGATL5* genes. In addition, we showed that *BnABI3* expression rescued *gs1* freezing-induced green seed coloration by targeting *AtSGR1/2* in transgenic *Arabidopsis*. *BnABI3* is also involved in lateral root development and conferred a novel interaction between ABA and auxin signaling in roots. The potential role of *ABI3* protein in endoplasmic reticulum homoeostasis was also tested. Altogether, our results indicated that *BnABI3* mediates both plant development and the stress response.

## Introduction

The ABA hormone participates in seed lipid and storage protein synthesis, seed desiccation tolerance and dormancy promotion as well as the inhibition of transition from embryonic to germinative growth (Rohde et al., 2000b). ABA signaling at these development stages is associated with the expression of several major regulatory genes involved in seed maturation. These regulatory genes, which exhibit somewhat redundant functions, include *LEC1* (*LEAFY COTYLEDON1*), *ABI3* (*ABSCISIC ACID INSENSITIVE3*), and *FUS3* (*FUSCA3*). *ABI3* is very conserved among the seeds of various plant species, such as tomato, wheat, maize, rice, carrot, oat and *Arabidopsis* (Nambara et al., 1992; Rohde et al., 1998; Suzuki et al., 2001; Shiota et al., 2006; Takenaka et al., 2007). ABI3 is a B3 domain-containing family protein and functions in the ABA signaling pathway in developing seeds. All of the ABI3 proteins have four highly conserved domains: an A1 transcriptional activation domain and three basic domains B1, B2, and B3 (Giraudat et al., 1992). The A1 domain shows the lowest similarity to orthologous in other plant species, but the B3 domain presents the highest similarity, exhibiting greater than 90% identity. The ability of ABI3 genes to activate ABA downstream responsive gene expression in seeds and embryos has been shown not only through transient gene expression experiments *in vitro* but also *in vivo* in expression assays in many different systems (Parcy et al., 1994; Parcy and Giraudat, 1997). The ABI3 transcription factors have important roles in the control of ABA-responsive genes in seed, especially those genes important for dormancy inception, desiccation tolerance and reserve deposition (Giraudat et al., 1992; Nambara et al., 1995; Rohde et al., 2000a; Shiota and Kamada, 2000; Zhang et al., 2005; Khandelwal et al., 2010; Finkelstein, 2013; Nakashima and Yamaguchi-Shinozaki, 2013).

Until now, many *Arabidopsis abi3* mutant alleles have been reported, among which the *abi3-6* mutant was initially identified through analyzing the stay-green phenotype of the seed. *abi3-6* seeds display ABA insensitivity and desiccation intolerance, and they often germinate prematurely (Giraudat et al., 1992; Ooms et al., 1993). Mutants with defective *ABI3* genes show disruption of developmental processes and altered transactivation of post-germinative related genes (e.g., maize malate synthase and isocitrate lyase genes, or *Arabidopsis* chlorophyll a/b binding genes) (Nambara et al., 2000; Rohde et al., 2000a). Additionally, less severe *ABI3* gene mutations affect developmental gene expression. For instance, a point mutation in the B2 domain of the ABI3 gene strongly down-regulates the expression of Em and albumin storage protein mRNA levels (Bies-Etheve et al., 1999).

Abscisic acid is an important phytohormone that has key roles in stress resistance and plant growth (Baron et al., 2012; Skubacz et al., 2016). A previous study suggested that cold stress was accompanied by increased levels of endogenous ABA (Mantyla et al., 1995), and exogenous ABA treatment could enhance plant cold resistance (Huang et al., 2015). Under low temperature, plants activate downstream gene expression through both ABA-dependent and ABA-independent pathways. The expression of ABA-responsive transcription factor genes *ABF1* and *ABF4* were up-regulated after cold treatment in *Arabidopsis* (Choi et al., 2000). Moreover, ABA treatment also up-regulated the soluble sugar content, improved enhanced water retention, reduced membrane lipid peroxidation and promoted photosynthesis (He and Li, 2008; Huang et al., 2015).

In this study, we have identified an *Arabidopsis* line (*gs1*) from a population of mutagenized seeds and showed that GS1 is a new allele of *AtABI3*. Then, we functionally characterized the *Brassica napus BnABI3*, which is an orthologous of the *ABI3* gene in *Arabidopsis*. The *BnABI3* gene could complement *gs1* seed phenotypes, and its overexpression rescued the seed coat defect, freezing-induced green seed coloration and increased freezing tolerance. The potential role of *BnABI3* in ER homoeostasis and LR development was elucidated, and a novel interaction of *BnABI3* and auxin in root growth was identified. These results indicate that *BnABI3* mediates both developmental progress (seed and LR development) and environmental responses (freezing tolerance and ER stress).

## Materials and Methods

### Plant Materials and Growth Conditions

*Brassica napus* L. (cv. HuYou15) seeds were obtained from the Shanghai Academy of Agricultural Sciences (Shanghai, China). The seeds were vernalized on wet filter paper in the dark for 1 month at 4°C. The germinated seedlings were transferred to soil in growth chambers under a 16-h/8-h (day/night) cycle at 22 ± 2°C. Col-0 ecotype *Arabidopsis thaliana* L., Heynh. was used in our study, and the *Arabidopsis* seeds were surface sterilized (Lindsey et al., 2017). Plates holding the seeds were then maintained in the dark at 4°C for 3 days to synchronize germination, and the seeds were subsequently planted in MS medium under a 16-h/8-h (day/night) cycle.

### Plasmid Constructions and Transgenic Plants Generation

*BnABI3* cDNA was cloned from *B. napus* L. (cv. HuYou15). *BnABI3* cDNA was sub-cloned into the pHB vector to generate 35S::*BnABI3* transgenic lines (Supplementary Figure S1) (Mao et al., 2005; Xu et al., 2016). To generate the proAtABI3::*AtABI3* and proAtABI3::*BnABI3* transgenic plants, 1.6 kb *AtABI3* promoter was fused with the *AtABI3* and *BnABI3* CDS sequences and sub-cloned into the pCAMBIA1300 vector. Transgenic *gs1* mutant expressing proAtABI3::*BnABI3* chimeric gene was obtained by hybridization. To generate 4Enhp BnABI3-BnABI3GR, the CaMV 35S enhancer tetrad was amplified using pSKI015 as template and cloned into pQDL4R1 to generate pQDL4R1-4Enh. The GR domain was cloned from pTA7002 and the coding region of *BnABI3* was cloned from cDNA. Both fragments were fused together and cloned into pQDR2L3 vector to generate pQDR2L3-BnABI3GR. All the reagents and digest enzymes used for PCR amplification and molecular clone were ordered from Takara, Japan. The constructs were transformed into *Agrobacterium tumefaciens* GV3101 and transformed into Col-0 *Arabidopsis* by using the floral-dip method (Clough and Bent, 1998). The positive transgenic plants were screened by 50 mg/L Hygromycin (Roche, United States). Real time PCR were performed to detect the gene expression levels in transgenic plants, and the primers used are shown in Supplementary Table S1.

### RNA Extraction and qRT-PCR Assay

Total RNA was isolated by using the RNeasy Plant Mini Kit (Qiagen, United States) and stored at -80°C. 1 μg total RNA was used for cDNA synthesis using the Transcript First-Strand cDNA Synthesis Mix (TianGen Bio-tech, China). According to the manufacturer’s instructions, qRT-PCR was performed in 96-well plates with 1 μl of a 1:5 dilution of the first-strand cDNA reaction and in a 10 μl volume on a Roche LightCycler^®^ 96 Sequence Detection System. Transcript levels were measured based on SYBR Green technology (Applied Biosystems Applera, Darmstadt, Germany). Data were analyzed by using LightCycler 96 analysis software 1.1 (ΔΔC_T_ method). *AtACT2* and *AtUBQ10* genes were used as internal controls (Xu and Cai, 2017). RT-qPCR primers are shown in Supplementary Table S2.

### DEX Induction Assay

The 5-week-old BnABI3GR transgenic plants siliques were treated with 20 μM DEX, 20 μM DEX plus 100 μM CHX, or DMSO (mock). The tissues were collected at the indicated time after treatments and put into liquid nitrogen immediately.

### Stress and Phytohormone Treatments

For each treatment, 4-week-old plants were transferred to Hoagland’s solution with 100 mM Mannitol, 100 mM NaCl, and 5 μM ABA for 48 h. The leaves sampled at each time point were obtained and stored at -80°C. For the freezing assay, plants were grown in soil in growth chambers at 21°C under long-day (LD) conditions for 3 weeks before the treatments were performed. For cold acclimation experiments, 3-week-old plants were acclimated for 3 days at 4°C in the light, then for 1 h at 0°C, before the temperature were decreased by 2°C per hour. The final sub-zero temperature was maintained for the indicated period before the temperature was again increased at the same rate to 4°C. The plants were then kept at 4°C for 1 day before they were returned to 21°C. Survival rate was counted 2 weeks later.

### Analyses of Seeds Total Proteins

Quantitative protein analyses were based on 40 seeds each. Total seed proteins, insoluble and buffer-soluble proteins were determined and separated according to a previous method (Bates et al., 2001). Different proteins were quantified using the Bio-Rad (Bio-Rad Laboratories, Hercules, CA, United States) Dc protein assay with three replicates.

### Quantification of Lipids

Mature seeds (50 mg) were analyzed for crude lipid content, and the method was carried out as described (Bates et al., 2001) with three replicates.

### Light Microscopy Observation

Mature dry seeds of Col-0, *abi3-6, gs1* and transgenic plants were fixed in PBS buffer containing 10% formaldehyde, 5% acetic acid, and 50% ethanol and subsequently dehydrated in a (10, 30, 50, 70, 80, 90,100% 1 h each step) graded ethanol series overnight. Seeds were then infiltrated with graded concentrations of LR White acrylic resin (Sigma, Co., St. Louis, MO, United States) at 25, 50, and 75%, each for a minimum of 3 h followed by two changes of 16 h 100% resin. Seeds were placed in fresh 100% LR White acrylic resin in an embedding capsule and polymerized at 60°C for 24 h, then observed under a light microscope (Leica, Germany).

### Scanning Electron Microscopy

The seeds were sputter-coated with gold and further visualized using a Hitachi JEOL JSM-6360LV SEM (scanning electron microscope).

### Seed Coat Permeability Assay

Tetrazolium red tests were used (Debeaujon and Koornneef, 2000; Debeaujon et al., 2000). Dried seeds of *Arabidopsis* were incubated in 1% (w/v) tetrazolium red (2, 3, 5-triphenyltetrazolium chloride, Sigma-Aldrich, United States) in the dark at 30°C for 24 h. The seeds were imaged by using a stereomicroscope.

### Transient Dual-Luciferase Reporter System

About 1.7-kb long promoter sequences of *AtMUM1, AtGATL5*, and *AtSGR1/2* were amplified from *Arabidopsis* genomic DNA and inserted into the plasmid reporter vector pGreen II 0800-LUC. The coding sequence of *BnABI3* was amplified via PCR and then inserted into the effector plasmid pGreen II 62-SK. *Arabidopsis* protoplasts were prepared and cotransfected with the constructs in accordance with the instructions for the Dual-Luciferase Reporter Assay system (Promega). The ratio of LUC to REN was determined for the dual-luciferase reporter system (Promega) via a Glomax 20/20 luminometer (Promega) after culturing the protoplasts under low-light conditions for 16 h. The LUN/REN ratio indicates the transcriptional activity.

### Chromatin Immunoprecipitation (ChIP) and qPCR

ChIP and subsequent qPCR (input DNA dilution of 1000×) were performed as previously described (Lavenus et al., 2015). The primers designed to amplify 110–180 bp fragments are listed in Supplementary Table S3. The relative enrichment of the target region was normalized to that of *AtACT2*. The relative enrichment of *BnABI3*-HA proteins was analyzed at the regions of target genes promoter. The 35S::*BnABI3*-HA transgenic line were analyzed via ChIP assays with anti-HA antibodies. The values were normalized to those of the internal controls.

### Statistical Analysis and Multiple Alignments

Statistical analysis was undertaken using the Mann–Whitney or Kruskal–Walls with GraphPad Prism 6.0 (GraphPad Software, San Diego, CA, United States). The differences were considered significant when *p* < 0.05. Multiple alignments and phylogenetic analyses were performed using DNA MAN 6.0 and MEGA 4.1 software (Hall, 2013).

## Results

### Green Seed 1 (GS1) Is a New Allele of ABI3 in *A. thaliana*

**FIGURE 1:**
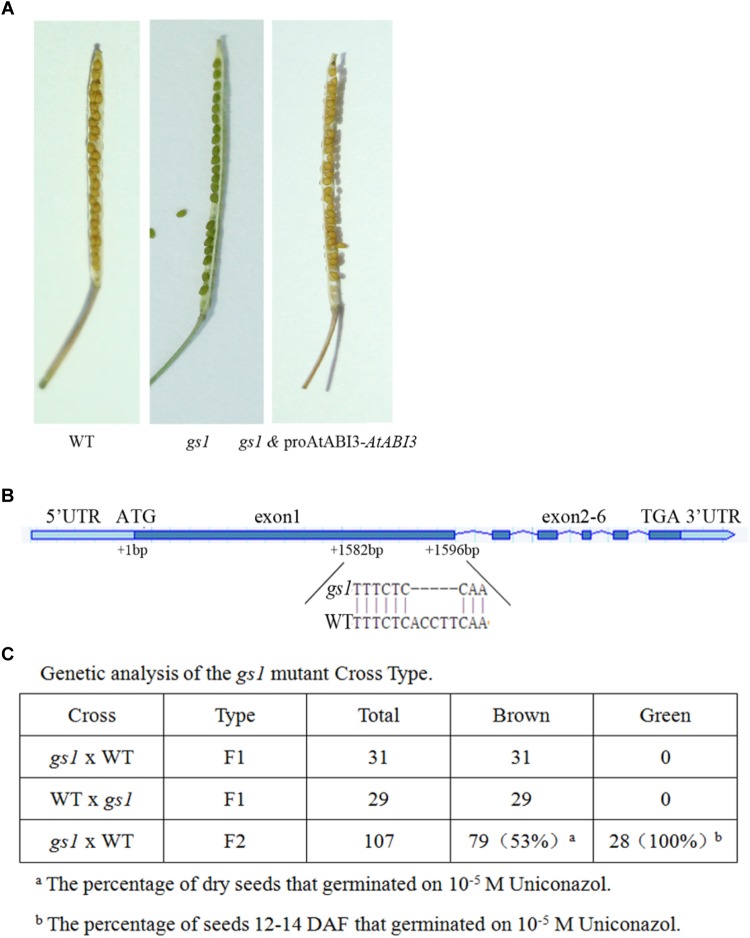
*gs1* is a mutant of new allele of the *AtABI3* in *Arabidopsis*. **(A)** Seed phenotype of the wild-type (WT), *gs1* mutant and a *gs1* complementary line (with the *AtABI3* gene fused with its native promoter). **(B)** The figure indicates the exons and introns of the *AtABI3* gene, and the *gs1* mutant harbors a 5 bp base deletion at the first exon. **(C)** Genetic analysis of the *gs1* mutant cross type and the percentage of seeds germinated on 10^-5^ M uniconazole.

Wild-type (WT) *Arabidopsis* seeds cannot be germinated on agar-solidified medium containing 10^-4^ M uniconazole (Nambara et al., 1991). We found the *Arabidopsis* line green seed 1 (*gs1*) from a population of approximately 9000 mutagenized M2 seeds, which germinated normally in media with 2 M uniconazole. In addition to the ability to germinate on 2 M uniconazole, the *gs1* mutant was segregated by the production of dark green shriveled seeds in the M3 generation, which could not be germinated after desiccation (Figure 1A). These seedlings produced green seeds that did not germinate when fully desiccated. The experiment of genetic segregation of the 107 M3 seeds showed that 28 seeds were green. This proportion fits the 3:1 hypothesis, suggesting that a recessive single gene mutation contributes to the green seed color phenotype. Reciprocal backcrosses were performed between the WT and *gs1* plants, and the data were consistent with the assumption that the green seed phenotype was the result of a monogenic recessive mutation. The genetic association between the green seed phenotype and the ability to germinate on uniconazole was assessed. All the green seeds removed from immature siliques could germinate on uniconazole, indicating that the green seed phenotype and the ability to germinate on uniconazole are due to the same gene mutation (Figure 1C).

The phenotype of *gs1* mutant seed is resulted from a single gene mutation and this abnormal seed phenotype is quite similar to the *abi3* mutant (Koornneef et al., 1989). Therefore, to identify whether the GS1 mutation is a new allele of the *abi3* mutant, we sequenced the entire ABI3 gene and found that a 5 bp deletion in the first exon of *AtABI3* which results in a truncated 385 aa protein (Figure 1B and Supplementary Figure S2). Furthermore, we used the ABI3 native promoter fused with the ABI3 CDS sequence to complement the *gs1* mutant. Many transgenic *gs1* and proAtABI3::*BnABI3* lines were generated. After confirmation via qPCR, we selected the lines for further assays (Supplementary Figure S3). The results showed that all transgenic lines have a brown seed coat and acquired desiccation tolerance. Thus, our results demonstrated that GS1 is a new allele of *ABI3*.

### Characterization of the cDNA Encoding the Putative *BnABI3* Gene in *B. napus*

**FIGURE 2:**
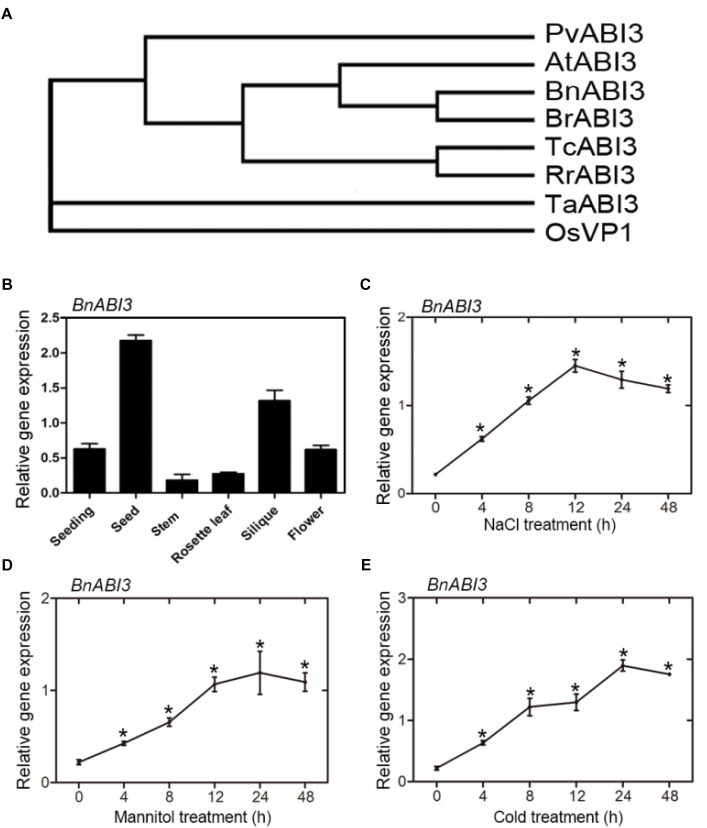
Phylogenetic tree analysis and gene expression patterns. **(A)** Multiple sequence alignment of the amino acid sequence of BnABI3 (LOC106361788) with sequences from other species: *Arabidopsis thaliana* (At3g24650); Rice (LOC_Os01g68370); *Phaseolus vulgaris* (XP_007156282); *Ribes rubrum* (KX061754); *Triticum aestivum* (AB085836); *Theobroma cacao* (XM_007049795); and *Brassica rapa* (XM_009137834). Protein sequences were aligned using the CLUSTALW alignment algorithm. A rooted phylogenetic tree based on sequence alignment using MEGA 5.1 software from CLUSTALW multiple sequence alignment. **(B)** Organ-specific expression of the *BnABI3* gene. Quantitative RT-PCR analyses were performed using total RNA extracted from the seeds, stems, leaves, siliques, flowers, and seedlings. Effects of **(C)** NaCl, **(D)** mannitol, and **(E)** cold stress on the levels of *BnABI3* mRNA in leaves. The error bars represent the SD. The presented values are the mean ± SD (*n* = 3). ^∗^*p* < 0.05 by Mann–Whitney test.

Employing primers generated using GenBank Accession No. LOC106361788 as a reference, we cloned a 2157 bp cDNA encoding a 718 aa polypeptide from the cDNA of *B. napus* L. (cv. HuYou15) leaves and identified a candidate *BnABI3* gene after DNA sequencing. Homology analysis indicated that BnABI3 was 65% identical to *A. thaliana* AtABI3 (Supplementary Figure S4). The phylogenetic tree was used to examine the evolutionary relationship between BnABI3 and ABI3 proteins derived from other species. These results showed that the ABI3 sequences of higher plants could be classified into two groups. The first group was composed of sequences from monocotyledonous plants, such as *Triticum aestivum* and rice, while the other group was composed of sequences from dicotyledonous plants, such as *A. thaliana, B. napus, Phaseolus vulgaris*, and *Ribes rubrum*. These results show that *BnABI3* is more closely related to the ABI3 genes of dicots than that of monocots (Figure 2A).

*BnABI3* gene expression levels in different plant stages and tissues, such as seedlings and flowers, leaves, stems, siliques, and seeds at different development stages, were assayed using qRT-PCR. The results showed relatively high expression levels of *BnABI3* in seeds and siliques, whereas lower expression levels were observed in stems (Figure 2B). To determine whether the expression of *BnABI3* is controlled by environmental cues, the mRNA levels of *BnABI3* were measured in the leaves of 3-week-old *B. napus* plants subjected to various abiotic stresses for a certain period. In response to NaCl treatment, early induction of *BnABI3* was observed at 4 h, with greater increases being detected at 12 h (Figure 2C). *BnABI3* gene expression gradually increased at 4 h after mannitol treatment, reaching the highest expression level at 24 h (Figure 2D). Maximum induction of *BnABI3* was observed after cold treatment for 24 h, with a reduction being detected at 48 h in leaves (Figure 2E). Taken together, these results showed that *BnABI3* responds to various environmental cues.

### Analysis of the proAtABI3::*BnABI3* Transgene in the *gs1* Mutant Background During Seed Germination and Post-germination Growth

**FIGURE 3:**
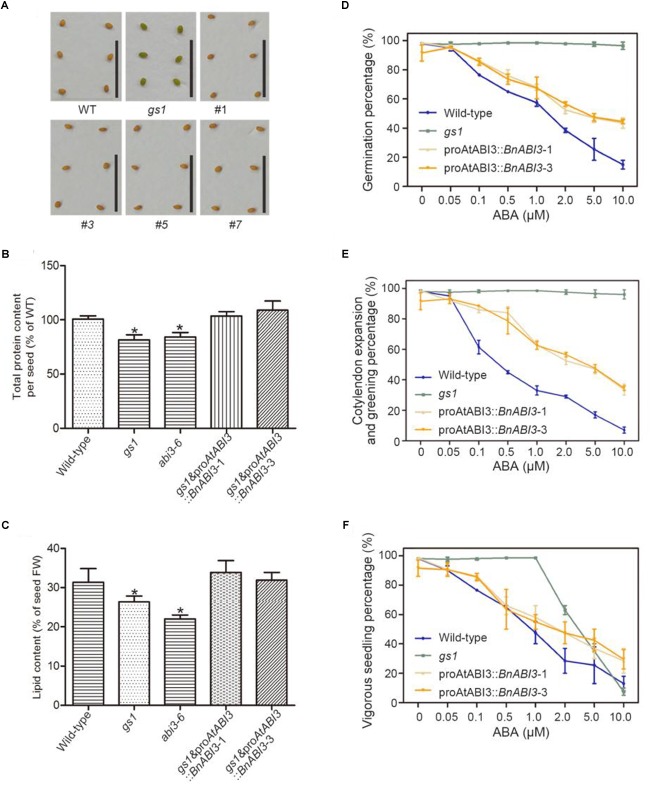
Analysis of protein, lipid contents, effect of exogenous ABA on germination and post-germinative growth of WT, *gs1* and *gs1* and proABI3::*BnABI3* transgenic plants. **(A)** Mature desiccated seeds of WT, *gs1*, and *gs1* and proABI3::*BnABI3* transgenic plants #1, #3, #5, #7. All images are at the same magnification. Bar indicates 1 cm. **(B)** Total protein content (WT as 100%) and **(C)** lipid content (% of FW) in WT, *gs1*, and *gs1* and proABI3::*BnABI3* transgenic plants **(D)** Percentage of germination at day 5 after various concentrations of ABA treatment. **(E)** Percentage of germinates showing cotyledon expansion and greening at day 5 after various concentrations of ABA treatment. **(F)** Percentage of germinants showing vigorous growth at day 18 after various concentrations of ABA treatment. ABA was applied to MS medium plates at the beginning of the moist chilling period, and the plates were transferred to germination conditions to monitor the percentage of germination as well as the growth characteristics of the germinants. Approximately 40 seeds were used in each treatment, and triplicate treatments were carried out. Germination percentages are based on radicle emergence. Values are given as mean ± SD, *n* = 4. ^∗^*p* < 0.05 by Kruskal–Wallis test.

Because *B. napus* is very difficult to transform, to identify whether *BnABI3* could complement the *gs1* mutant, we constructed a series of *Arabidopsis* transgenic lines carrying a proAtABI3:: *BnABI3* gene sequence in a Col-0 background and then crossed these lines with *gs1* mutant plants (Supplementary Figure S1). To fix the transgene in a homozygous *gs1*, brown seeds were selected from the basta-resistant plants showing about a 3:1 segregation of seed color. The selected F3 plants were expected to be homozygous for *gs1* and hemizygous for the transgene. From this assay, we concluded that the exogenous proAtABI3::*BnABI3* transgene was able to complement the *gs1* mutant seed coat phenotype (Figure 3A). We also observed protein and lipid content in the *BnABI3* transgenic seeds. Compared with *gs1* and *abi3-6* mutants, there was an obvious difference in the total protein content of the *BnABI3* transgenic seeds. Transgenic seeds exhibited the full complement of storage proteins expressed at Col-0 levels (Figure 3B). Moreover, there was a reduced lipid content in *abi3-6* and *gs1* seeds, and *BnABI3* gene expression can absolutely restore the lipid content of *abi3* mutants to Col-0 levels (Figure 3C).

The *abi3-6* plants are completely insensitive to ABA treatment at the germination stage (Giraudat et al., 1992). Hence, we wanted to determine whether proAtABI3::*BnABI3* lines can restore ABA sensitivity. The exogenous ABA biological effects on seed germination and plant growth were further assayed. After 4 days of moist chilling treatment, all seeds showed almost an equal ability to germinate on the media without ABA. As expected, the germination of the Col-0 seeds was inhibited in a gradient of ABA-containing media, but the *gs1* mutant seeds displayed no sensitivity up to 10 mM ABA. The dose-response curves of transgene seeds on ABA-containing media were more similar to the Col-0, suggesting that transgene lines partly restored the *gs1* mutant ABA sensitivity (Figure 3D). The results also showed that seeds of the *gs1* mutant were highly insensitive to ABA and exhibited almost 100% germination. The seeds of WT plants were the most sensitive to ABA, and their germination was increasingly inhibited by 1–10 mM ABA. At ABA concentrations greater than 2 mM, most of the Col-0 plants could not expand their cotyledons and develop into normal seedlings. Interestingly, the seeds of all three transgenic plants showed ABA sensitivities that were intermediate to those of the *abi3* mutants (*gs1*) and Col-0 (Figure 3E,F). The somewhat different sensitivity to ABA during post-germinative growth may show the inability of the BnABI3 protein to completely restore ABA sensitivity in the *gs1* mutant background.

### *ABI3* Regulates Seed Coat Development

**FIGURE 4:**
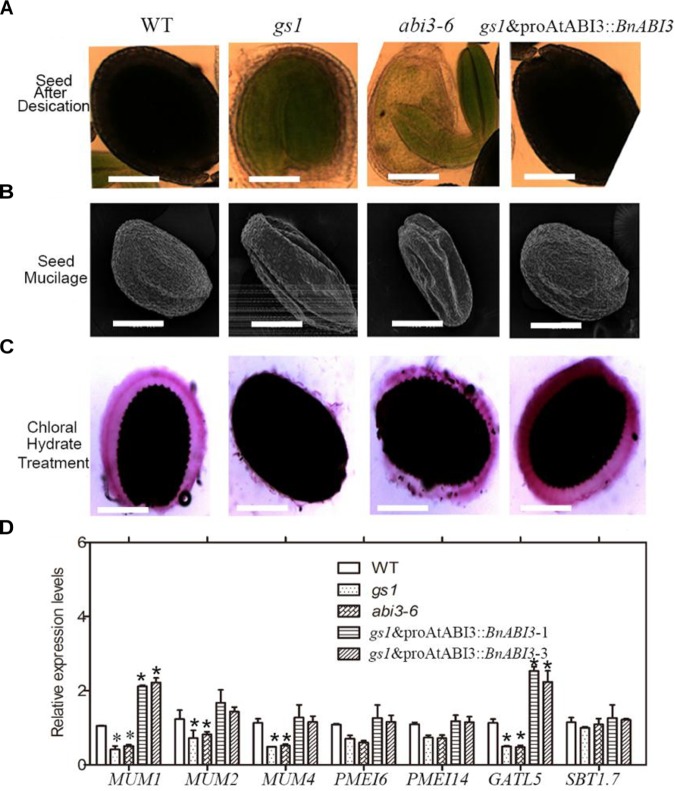
The *ABI3* regulates seed coat development. **(A)** Analysis of transparency in mature seeds from Col-0, *gs1*, and *abi3-6* mutants and *gs1* and proAtABI3::*BnABI3* plants after treatment with chloral hydrate liquid. **(B)** Mature seeds from Col-0, *gs1*, and *abi3-6* mutants and *gs1* and pAtABI3::*BnABI3* plant seeds after 3 days desiccation treatment in the 37°C drying oven. **(C)** Observation of seed coat mucilage based on staining with Ruthenium red. **(D)** RT-qPCR analysis of *MUM1, MUM2, MUM4, PMEI6, PMEI14, GATL5*, and *SBT1.7* gene expression in Col-0, *gs1, abi3-6* mutant, and proAtABI3::*BnABI3* transgenic plant seeds. ^∗^*p* < 0.05 by Kruskal–Wallis test.

Research in the desiccation tolerance of plant seeds has immense basic and applied value (Angelovici et al., 2010). Revealing the responses and adaptations of seeds to rigorous desiccation is essential to improve commercial crop plants by developing these characteristics (Joet et al., 2013). Although *ABI3* has important roles in seed desiccation tolerance (Zeng et al., 2003), the underlying molecular mechanism is not well-understood. Previous studies have shown that *ABI3* regulates the accumulation of storage proteins involved in desiccation tolerance (Nambara et al., 1992; Khandelwal et al., 2010). However, in the present study, we showed that *BnABI3* can regulate seed coat development, thereby playing a role in seed desiccation tolerance. As shown in Figure 4A, we used chloral hydrate to achieve transparency in the premature seeds at 16 days after flowering (DAF) and found that transparency was easily achieved in the *gs1* and *abi3-6* mutant seed coats at this stage after 3 min, and the seed coats were fragile after treatment (could be broken with moderate pressure). In addition, after storage at 37°C for 24 h, the mature WT seeds appeared normal, but the *gs1* and *abi3-6* mutant seeds appeared hollow (Figure 4B). Furthermore, we monitored mucilage secretion and found that the *gs1* and *abi3-6* mutants were defective in this process (Figure 4C). However, this phenotype was not found in the Col-0 seeds, and *gs1* and proAtABI3::*BnABI3* transgenic plants could fully complement the mutant phenotype in the *gs1* background. Thus, the *ABI3*-mediated intact seed coat could at least partially contribute to seed desiccation tolerance.

### *BnABI3* Regulates Mucilage Secretion by Directly Targeting *AtMUM1* and *AtGATL5* Genes

During differentiation, the epidermal cells of *Arabidopsis* seed coat produce and secrete large quantities of mucilage, making it a valuable model for studying cell wall biology. Mucilage is composed mainly of pectin, and also contains cellulose, hemicellulose, etc. By using RT-qPCR analyses, we checked the expression levels of mucilage secretion related genes such as *MUM1, MUM2, MUM4, PMEI6, PMEI14, GATL5*, and *SBT1.7* in Col-0, *abi3* mutants and proAtABI3::*BnABI3* transgenic plants (Shi et al., 2018) (Figure 4D). We found that the expression levels of *MUM1* and *GATL5* were altered obviously. The mucilage of the *mum1* mutant contains rhamnogalacturonan I that is more highly branched and lacks the ability to be extruded when exposed to water (Arsovski et al., 2009; Francoz et al., 2015). *GATL5* is involved in mucilage synthesis and may function to regulate the size of the polysaccharide (Kong et al., 2013).

**FIGURE 5:**
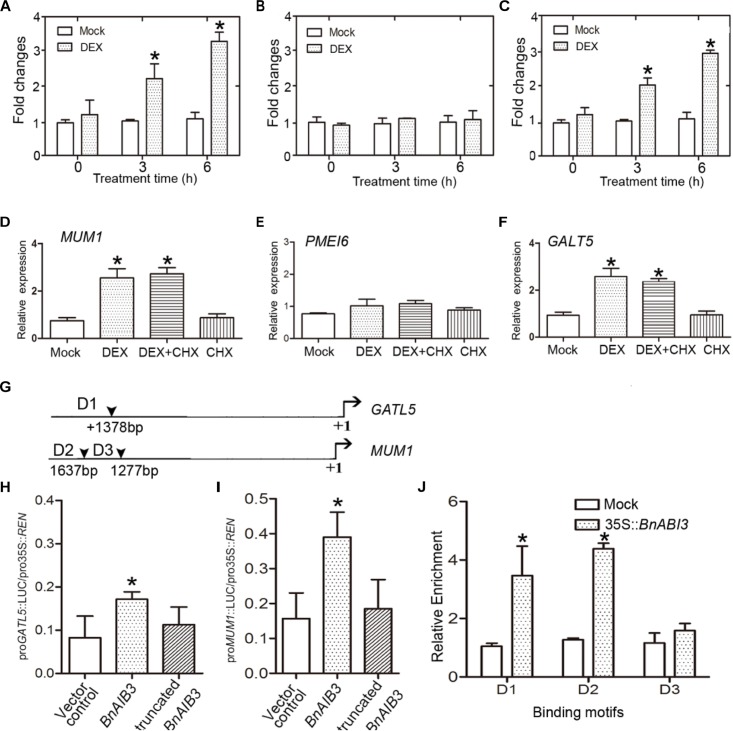
*BnABI3* regulates mucilage secretion by targeting *MUM1* and *GALT5* expression. **(A–C)** Relative expression of *MUM1, PMEI6*, and *GALT5* in BnABI3GR transgenic plant siliques treated with 20 μM DEX or mock for 0, 3, or 6 h. The expression of the corresponding genes in mock-treated plants was set to 1.0. Values are given as mean ± SD, *n* = 3. ^∗^*p* < 0.05 by Mann–Whitney test. **(D–F)** Relative expression level of *MUM1, PMEI6*, and *GALT5* in BnABI3GR transgenic plant siliques treated with 20 μM DEX, 100 μM CHX, 20 μM DEX plus 100 μM CHX, or mock. The gene expression in mock-treated plants was set at 1.0. Values are given as mean ± SD, *n* = 3. ^∗^*p* < 0.05 by Kruskal–Wallis test. **(G)** Schematic diagram indicating the locations of ABI3 binding sites (D1 to D3) in the *GALT5* and *MUM1* genes promoters. **(H,I)** Transient dual-luciferase reporter assay. The pGreenII-0800 luciferase (LUC) construct containing the *GATL5* and *MUM1* promoters and the p62-SK construct with the *BnABI3* coding region and truncated *BnABI3* without DNA binding domain were transiently co-transformed into Col-0 protoplasts. Firefly LUC and Renilla luciferase (REN) activities were measured after culturing the protoplasts under low-light conditions for 16 h. Values are given as mean ± SD, *n* = 4. **(J)** ChIP-qPCR analysis of the ability of BnABI3 to bind to the promoters of *GATL5* and *MUM1*. An anti-HA monoclonal antibody was used for DNA immunoprecipitation from 2-week-old 35S::*BnABI3*-HA transgenic plants. Gray bars indicate the enrichment fold changes normalized to ACT2. Values are given as mean ± SD, *n* = 3. ^∗^*p* < 0.05 by Kruskal–Wallis test.

To investigate the mechanisms underlying the function of *BnABI3* in mucilage secretion biosynthesis, we wanted to determine whether *MUM1* and *GATL5* were immediately downstream of *BnABI3* since they were induced in BnABI3GR transgenic plants after dexamethasone (DEX) induction in the presence of cycloheximide (CHX) (Figure 5A–F). The DEX induction assays suggested that *MUM1* and/or *GATL5* were likely to be downstream targets of BnABI3, which contributes to altered mucilage biosynthesis. Furthermore, we analyzed the promoter sequences of both *MUM1* and *GATL5* genes. The CATGCA core sequence was reported to be crucial for ABI3 transcription factor binding. There are two and one ABI3 binding motifs in the *MUM1* and *GATL5* promoter sequences (Figure 5G). We used a LUC-based transactivation experiment. We co-expressed *BnABI3* with both *MUM1* and *GATL5* promoter-LUC fusion reporters in mesophyll protoplasts of *Arabidopsis*, which resulted in an increase in luciferase activity by 2.4- and 2.7-fold (Figure 5H,I). To provide further evidence for the interaction, ChIP assays were conducted to identify whether the BnABI3 protein could bind to the gene promoters. We used 35S::*BnABI3*-HA transgenic plants, which contained an HA-coding sequence fused in-frame to the 3′ end of the *BnABI3* gene. ChIP-qPCR assays using an anti-HA antibody showed that BnABI3 could bind to the conserved sequence motifs in the *MUM1* and *GATL5* gene promoters (Figure 5J). These results demonstrate that BnABI3 could target the *MUM1* and *GATL5* gene promoters and induce their transcription.

### *BnABI3* Regulates Freezing-Induced Green Seed Coloration by Targeting *AtSGR1*/*2* Genes

**FIGURE 6:**
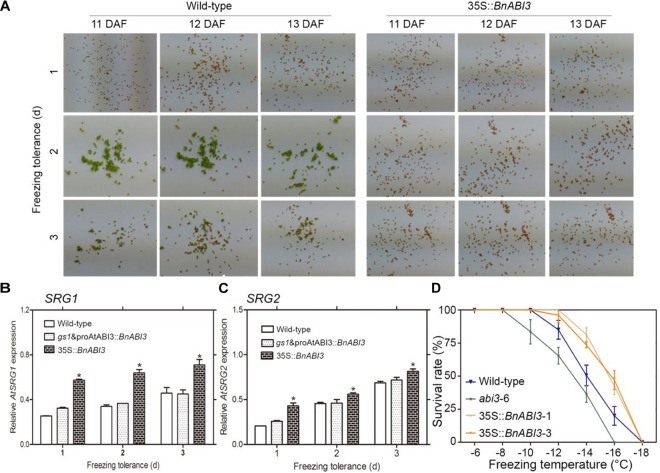
*BnABI3* overexpression rescues the freezing-induced green seed phenotype in *Arabidopsis*. **(A)** Mature seeds from Col-0 and 35S::*BnABI3*-1 plants showing the freezing-induced effect on de-greening. *Arabidopsis* plants were exposed to temperatures of –5 to –10°C (2 h/day) for 1–3 days at various stages of silique development, followed by maintenance at 22°C temperature. After maturation, the seeds were harvested and examined for green seeds. **(B,C)** RT-qPCR analysis of *SGR1* and *SGR2* expression in seeds (11–13 DAF) either left untreated or exposed to freezing and allowed to recover for 2 days at ambient temperature (*n* = 4). **(D)** Survival rate analysis of WT (Col-0), *gs1* and proAtABI3::*BnABI3* and 35S::*BnABI3* transgenic plants after –6 to –18°C freezing treatment. Values are given as mean ± SD, *n* = 4. ^∗^*p* < 0.05 Kruskal–Wallis test.

Frost is one of the major factors that can cause the fixation of a green color in mature seeds. Exposure of plants from 0 to 1°C sub-lethal frost can fix a green color in seeds, which contributes to major seed industry losses (Johnson-flanagan et al., 1994; Jalink et al., 1998). Because ABI3 controls embryo degreening through Mendel’s I locus (Delmas et al., 2013), we hypothesized that if freezing stress resulted in the down-regulation of chlorophyll degradation genes, *BnABI3* overexpression should help to overcome this problem. To verify this hypothesis, we investigated maturing of *Arabidopsis* pods at 11–13 DAF and found that freezing temperatures resulted in mature green seeds (Figure 6A). However, when 35S::*BnABI3* transgenic lines were subjected to similar treatment, the transgenic seeds proceeded to de-green and produced mature brown seeds (Figure 6A). qRT-PCR analyses showed that *BnABI3* over-expressing lines expressed much higher levels of *SGR1* and *SGR2* compared with Col-0 after freezing treatment (Figure 6B,C).

**FIGURE 7:**
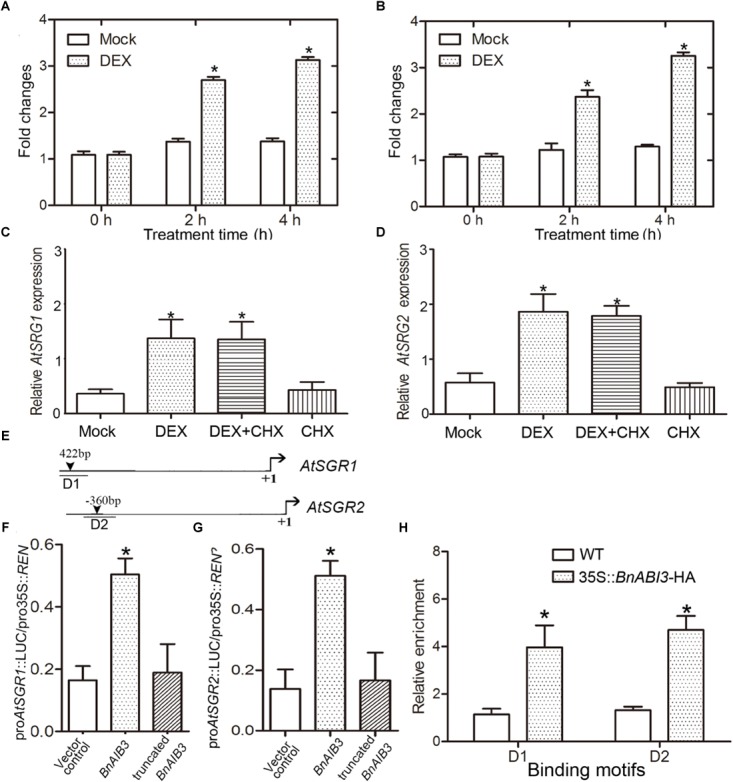
BnABI3 targets *AtSGR1*/*2* genes. **(A,B)** Relative expression of *SGR1* and *SGR2* in BnABI3GR transgenic plants treated with 20 μM DEX or mock for 0, 2, or 4 h. The expression of the corresponding genes in mock-treated plants was set to 1.0. Values are given as mean ± SD, *n* = 3. **(C,D)** Relative expression level of *SGR1* and *SGR2* in BnABI3GR transgenic plants siliques treated with 20 μM DEX, 100 μM CHX, 20 μM DEX plus 100 μM CHX, or mock. The gene expression in mock-treated plants was set to 1.0. Values are given as mean ± SD, *n* = 3. **(E)** Schematic diagram indicating the locations of two ABI3 binding sites (D1 to D2) in the *SGR1* and *SGR2* gene promoters. **(F,G)** Transient dual-luciferase reporter assay. The pGreenII-0800 LUC construct containing the *BnABI3* promoter and the p62-SK construct with the *BnABI3* coding region and truncated *BnABI3* without DNA binding domain were transiently co-transformed into Col-0 protoplasts. Firefly luciferase (LUC) and Renilla luciferase (REN) activities were measured after culturing the protoplasts under low-light conditions for 16 h. Values are given as mean ± SD, *n* = 4. **(H)** ChIP-qPCR analysis of the ability of BnABI3 to bind to the promoters of *SGR1* and *SGR2*. An anti-HA monoclonal antibody was used for DNA immunoprecipitation from 35S::*BnABI3*-HA transgenic plants. Values are given as mean ± SD, *n* = 4. ^∗^*p* < 0.05 by Mann–Whitney test.

Furthermore, we wanted to determine whether the expression levels of the altered genes in 35S::*BnABI3* plants were immediate downstream targets of BnABI3. DEX induction coupled with CHX treatment assays showed that *AtSGR1/2* was immediately up-regulated after DEX induction in the BnABI3GR transgenic plants (Figure 7A–D). Furthermore, both promoters possessed the canonical B3 domain binding RY motif CATGCA with variable flanking nucleotides (Figure 7E). We co-expressed *BnABI3* with *AtSGR1/2* promoter-LUC fusion reporters in mesophyll protoplasts of *Arabidopsis*, which resulted in an increase in luciferase activity by 3.1 and 3.4-folds (Figure 7F,G). ChIP assays were conducted using 35S::*BnABI3*-HA transgenic plants to identify whether the BnABI3 protein binds to the *AtSGR1/2* promoters. ChIP-qPCR assays using an anti-HA antibody indicated that BnABI3 could bind to the selected regions in the AtSGR1/2 promoters (Figure 7H). Taken together, we concluded that positive regulation of the *AtSGR1/2* genes by *BnABI3* is mediated mainly by increased transcription.

The freezing-induced green seed phenotype was rescued in *BnABI3* transgenic plants, suggesting that the exogenous expression of *BnABI3* might improve freezing tolerance at the seedling stage. To examine this possibility, we compared the development of freezing tolerance after cold acclimation. The *BnABI3* transgenic plants acclimated more quickly than the WT plants, while a lag before freezing tolerance started to increase in the *abi3* mutants, so the maximum tolerance achieved in the *BnABI3* transgenic plants was higher than in the WT (Figure 6D). We checked the expression profiles of *SRG1* and *SRG2* genes during cold acclimation in WT and *BnABI3* plants rosette leaves. We found that *SRG1* and *SRG2* genes expressions increased to higher level than that in WT control during cold acclimation. We also identified the freezing tolerance without cold acclimation; the results showed that *BnABI3* overexpression also increased freezing tolerance without cold acclimation (Supplementary Figure S5). Overall, we showed that *SRG1* and *SRG2* could play a role in *BnABI3* promoted freezing tolerance in transgenic *Arabidopsis.*

### *BnABI3* Regulates Flowering Time

**FIGURE 8:**
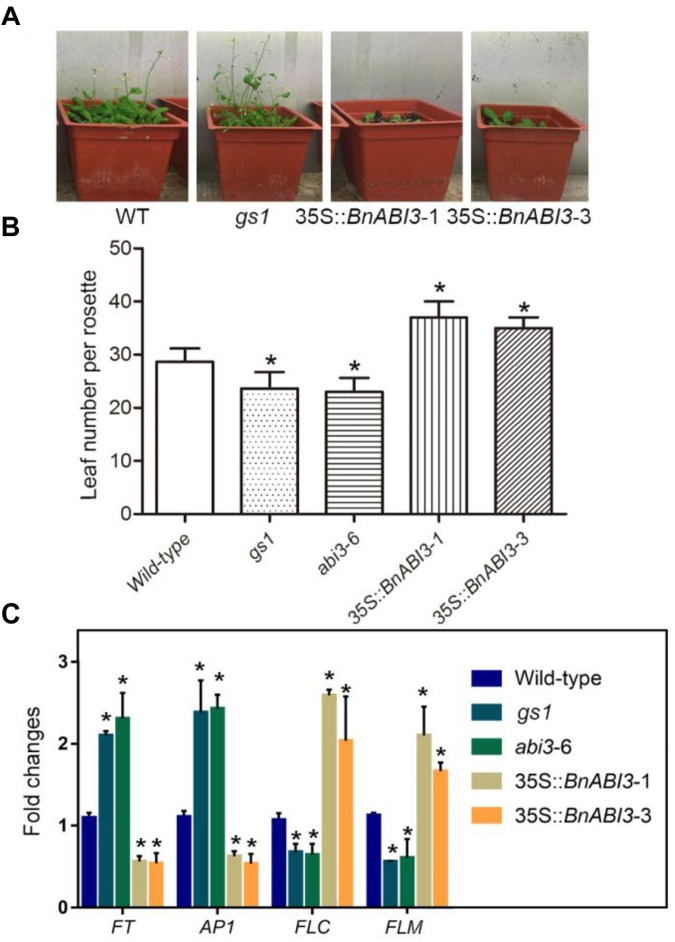
Flowering time and RT-qPCR analysis of flowering time related genes’ expressions in WT, *abi3* and transgenic *Arabidopsis* plants. **(A)** Flowering of WT and transgenic plants. **(B)** Number of rosette leaves in WT, *gs1, abi3-6*, and 35S::*BnABI3* transgenic *Arabidopsis* plants at bolting. The data represent the mean ± SD of three independent experiments (14 seedlings). **(C)** RT-qPCR analysis of the expression of the flowering time genes *FT, AP1, FLC*, and *FLM* in WT and transgenic *Arabidopsis* seedlings. Values are given as mean ± SD, *n* = 4. ^∗^*p* < 0.05 by Kruskal–Wallis test.

Because the previous study showed that the *abi3* mutant exhibits early flowering. Hence, we calculated the 35S::*BnABI3* plants flowering time to determine whether *BnABI3* regulated the flowering time. The results showed that *BnABI3* overexpression delayed flowering time (Figure 8A). There were more rosette leaves at bolting in the 35S::*BnABI3* transgenic lines compared with WT (Figure 8B). We also analyzed mRNA level of the flowering time related genes *FT, AP1, FLC*, and *FLM. FT* and *AP1* showed down regulated expression levels in the 35S::*BnABI3* transgenic plants. But *FLC* and *FLM* mRNA level increased in 35S::*BnABI3* transgenic plants (Figure 8C). The altered expression levels of flowering gene in 35S::*BnABI3* plants may contribute to the late flowering phenotype.

### *BnABI3* Plays a Role in the ER Stress Response

**FIGURE 9:**
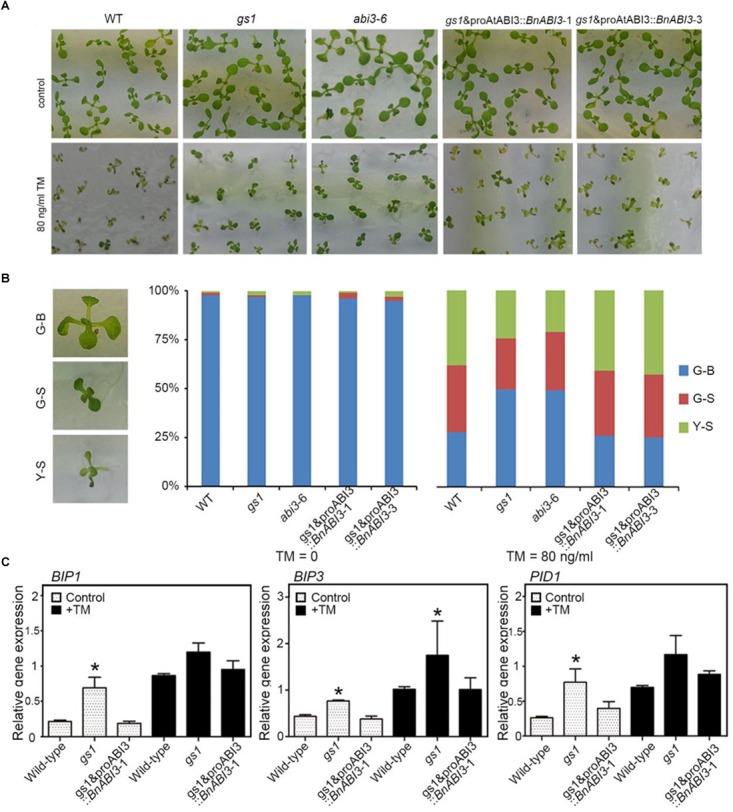
*BnABI3* overexpression confers ER stress tolerance. **(A)** The sensitivity of WT, *gs1*, and *abi3-6* mutant and *gs1* and proAtABI3::*BnABI3* transgenic plants to ER stress. Photographs of 10-day-old *Arabidopsis* seedlings grown on MS medium supplied without or with 80 ng/ml tunicamycin (TM) are shown. **(B)** The percentage of Green-Big (G-B), Green-Small (G-S), and Yellow-Small (Y-S) plants were calculated. The difference between the WT and each transgenic line is shown. Bars depict SD (*n* = 3). **(C)** RT-qPCR analysis of *BIP1, BIP3*, and *PID1* expression levels in WT, *gs1*, and *abi3-6* mutant and *gs1* and proAtABI3::*BnABI3* transgenic plants were either left untreated or exposed to TM. A group of 10 plants was measured for each genotype. Values are given as mean ± SD, *n* = 3. ^∗^*p* < 0.05 by Mann–Whitney test.

In addition to investigating how *BnABI3* regulates freezing tolerance, we also investigated the biological function of the *ABI3* in the ER stress response. Our results showed that *AtABI3* down-regulation confers ER stress tolerance in *Arabidopsis*. Both *gs1* and *abi3-6* mutant plants grew as well as Col-0 under our normal growth conditions, but they were more tolerant to ER stress than WT in the medium added with the ER stress inducers tunicamycin (TM) (Figure 9A). Under ER stress conditions, a greater number of G-B (Greenish Big) plants and fewer Y-S (Yellowish Small) plants were observed in the *abi3* mutants than in the control (Figure 9B). Taking these findings together, we conclude that mutation of *AtABI3* in *Arabidopsis* promotes ER stress tolerance. Moreover, exogenous expression of the *BnABI3* gene under the *AtABI3* promoter decreased ER stress tolerance to WT levels (Figure 9A,B). Furthermore, the levels of the ER-resident molecular chaperone genes *BIP1, BIP3*, and *PID1* were up-regulated in *gs1* and *abi3-6* mutant seeds (Fernandez-Bautista et al., 2017; Iwata et al., 2018) (Figure 9C), and the up-regulated expression of *BIP* and *PDI* genes could improve ER stress tolerance. In addition, we investigate the expression levels of ER stress related genes by using *BnABI3*GR transgenic plants. As shown in Supplementary Figure S6, induced expression of *BnABI3* by DEX treatment down regulated the *BIP1, BIP3*, and *PID1* expression levels and result in downregulated stress tolerance. So, we get the conclusion that *BnABI3* contributed directly to ER-stress response.

### *BnABI3* Involved in LR Development and Confers a Novel Interaction of ABA and Auxin in Root Growth

**FIGURE 10:**
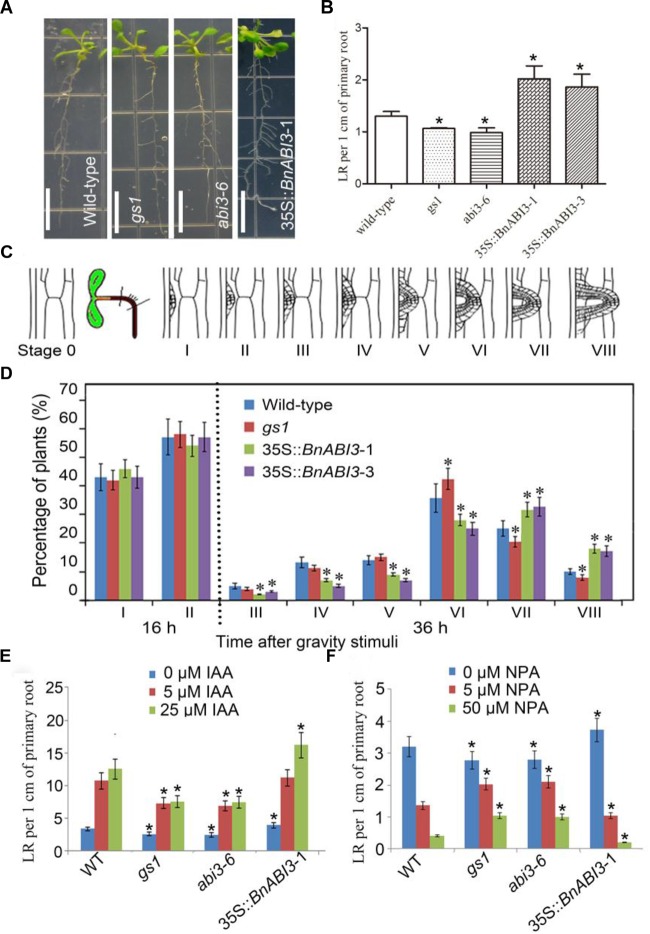
*BnABI3* is involved in lateral root (LR) development and confers a novel interaction of ABA and auxin signaling in roots. **(A)** Root phenotype and **(B)** LR per 1 cm primary root of 7-day-old WT, *gs1*, and *abi3-6* mutant and 35S::*BnABI3* transgenic plants. **(C)** After gravitropic stimulation to induce the synchronized initiation of LR primordia at the surface of bending roots, LR primordium stages from I to VIII. **(D)** Phenotypic analysis of LR emergence was achieved by synchronizing LR formation with a gravity stimulus for 16 and 36 h. Compared with WT (Col-0) the transgenic plants showed increased LR emergence. Data are shown as percentages, and the error bars represent SD, *n* = 11–15. At least 34 total LR primordia were observed for each plant. **(E,F)** LR per 1 cm primary root of 3-day-old WT, *gs1*, and *abi3-6* mutant and 35S::*BnABI3* transgenic plants exposed to various concentrations of IAA and NPA treatments for 10 days. Bar indicates 1 cm. Values are given as mean ± SD, *n* = 4. ^∗^*p* < 0.05 by Kruskal–Wallis test.

In addition, we want to identify whether *ABI3* plays roles in root development. The density of LR in the *gs1* and *abi3-6* mutants were decreased than that in Col-0, but the density of LR observed in 35S::*BnABI3* were increased than that in Col-0 (Figure 10A,B). Because, both mechanical and/or gravitropic stimuli can facilitate the initiation of LR. Previously, reports showed that LR initiation occurs in a highly synchronized manner at the outer surface of a bending root using 90° gravitropic stimulus assays (Peret et al., 2012; Voss et al., 2015). We can classify this developmental progress into many different stages (Figure 10C). In stage I, the LR primordia were observed at 16 h post-gravitropic-induction (pgi), until the LR emerged at 36 h pgi in stage VIII (Figure 10D). Next, WT, mutant and 35S::*BnABI3* plants were exposed to a gravitropic stimulus. WT plants accumulated stages I and II LRP at 16 h pgi and stages III to VIII at 36 h pgi. LR initiation and first divisions were not altered in the 35S::*BnABI3* plants but altered percentage of stages III–VIII LRP at 36 h pgi (Figure 10D). This result indicated that *BnABI3* overexpression affected LR emergence.

The ABA signaling pathway and auxin interact to regulate many different development processes (Brady et al., 2003; Nag et al., 2005; Rock and Sun, 2005). Therefore, we decided to identify the ability of *abi3* mutants and 35S::*BnABI3* plants root growth in the presence of IAA and the auxin transport inhibitor NPA (*N*-1-naphthylphthalamic acid) to search for any phenotypes that arise under conditions in which auxin-regulated LR development is perturbed. Under these growth conditions, *bs1* and *abi3-6* plants required higher concentrations of IAA to initiate LR growth, and 35S::*BnABI3* plants increased response to IAA treatment (Figure 10E). Similarly, application of the auxin transport inhibitor NPA inhibited LR initiation in both the WT and *abi3* plants; the mutant roots were still less responsive but 35S::*BnABI3* plants more response to NPA-induced repression of LR initiation (Figure 10F). Thus, it appears that ABI3 function is required for appropriate auxin responsiveness in the LRs of *Arabidopsis* plants.

## Discussion

Abscisic acid controls many biological processes, including the induction of stomatal closure, seed dormancy, root growth inhibition, and environmental stress response. Biochemical and molecular experiments have shown that the gene encoding the transcription factor ABI3 is one of the most important regulatory genes in the ABA signaling pathway for seed development in plants (Nambara and Marion-Poll, 2005). Here, we screened a new allele of the *Arabidopsis ABI3* gene, *gs1*, which allowed plants to germinate on 2 M uniconazole and caused them to produce green seeds. Furthermore, the cDNA encoding BnABI3 was isolated from *B. napus*. This putative BnABI3 protein showed well-conserved structural features (Nitsch et al., 2009). A complementary assay showed that *BnABI3* fused with the *AtABI3* native promoter could restore the *abi3* mutant green seed phenotype and seed desiccation tolerance (Figure 3A, 4B). Although there is a reduced accumulation of lipids in *gs1* mutant seed, expression of exogenous *BnABI3* resulted in a full complement of the lipid content to WT levels (Figure 3C). Interestingly, the seeds of 35S::*BnABI3* transgenic plants showed ABA sensitivity intermediate to that of the *gs1* mutant and WT (Figure 3D–F). The pro*AtABI3*::*BnABI3* seeds exhibited much higher sensitivity to exogenous ABA compared with that of the *gs1* mutant seeds in terms of germination. The differences in sensitivity during post-germinative growth may indicate an inability of the BnABI3 protein to restore ABA sensitivity completely in the *gs1* mutant. These differences may be due to the functional differences between the *Arabidopsis* ABI3 and its *B. napus* homolog protein.

The underlying molecular mechanism of desiccation tolerance is poorly understood. But whether or not the *ABI3* gene involved in seed coat development is still unknown. After careful observations, we found that the transparency of the *gs1* and *abi3-6* mutant-stage seed coats could be easily achieved using chloral hydrate, and the seed coat was rendered fragile after treatment (Figure 4A). Moreover, the *abi3* mutants exhibit defects in mucilage secretion processes (Figure 4C). The proAtABI3::*BnABI3* transgenic plants showed full complementation of the mutant phenotype in the *gs1* background. By using a LUC-based transactivation experiment and ChIP assay, we have indicated that BnABI3 could bind to conserved sequence motifs in the promoters of the mucilage secretion biosynthesis related genes *MUM1* and *GATL5* to induce their transcription levels (Figure 5G–J). For the first time, we report that ABI3 protein is involved in desiccation tolerance by directly regulating the development of the seed coat in addition to regulating storage proteins.

The presence of chlorophyll in mature seeds can affect seed maturation and oil quality. The occurrence of mature green seeds caused by low temperature during seed maturity in oil crops such as *B. napus* can lead to reduced income. One recently identified candidate that controls the chlorophyll degradation machinery is SGR1. *SGR1* mutations lead to stay-green leaf phenotypes in *Arabidopsis* and rice, but the signaling machinery that converges on SGR1 has remained elusive. The B3 domain transcription factor ABI3 has a highly conserved and essential role in seed maturation, conferring desiccation tolerance. Furthermore, we demonstrated that BnABI3 can directly target *SGR1/2* by binding to their promoters, and *BnABI3* gene overexpression was sufficient to rescue the cold-induced green seed phenotype in *gs1* and proAtABI3::*BnABI3* transgenic plants. These results imply that regulating *ABI3-SGR1/2* could resolve the green seed challenge in oil crops. Because the *ABI3* gene is the master regulator of de-greening (Delmas et al., 2013), and green seed problem is a major industry concern in oil-seed crops such as canola (Johnson-flanagan et al., 1994; Jalink et al., 1998), we found that the important role of ABI3 as the master regulator in this process is conducive to provide a way to tackle the green seed problem. Overexpression of *BnABI3* could provide a solution to the green seed problem associated with oil seeds.

Plants must integrate environmental cues and endogenous signals to fine-tune the timing of flowering. Low temperature is one of the main environmental factors affecting flowering time. Due to the expression pattern of *BnABI3*, we propose that it plays important roles in coordinating the cold response and flowering time. We also found the flowering time related genes *AP1* and *FT* were down-regulated, but *FLC* mRNA level increased in *abi3* mutants (Figure 8). The *FLC* transcription factor functions as a repressor of floral transition and contributes to the circadian clock temperature compensation. Our data also demonstrate that, even if the function of ABI3 is normally restricted seeds and control of seed-specific gene expression, it also has the ability to trigger activation of ABA-responsive genes whose expression is normally restricted to the vegetative tissues. Moreover, we observed that *abi3* mutant plants accumulate higher levels of ER stress response genes. We showed that a greater number of G-B plants and fewer Y-S plants occurred in the *gs1* and *abi3-6* mutants than in Col-0 under ER stress conditions (Figure 9A,B). In addition, the levels of the ER-related molecular chaperones genes *BIP* and *PDI* were up-regulated in the mutants (Figure 9C). Overall, both freeze sensitivity and enhanced ER stress in *abi3* mutants promote flowering time. Thus, we speculate that *BnABI3* serves as an important regulatory link between plant growth and environmental cues.

The possible mechanisms of crosstalk between ABA and auxin signaling in root growth are not well-understood. Studies of ABA and auxin have revealed that these pathways impinge on each other (Boerjan et al., 1995). The ABA-regulated AP2 domain transcription factor ABI4 was shown to repress expression of the auxin transporter PIN1, and *abi4* mutants display enhanced root auxin transport (Shkolnik-Inbar and Bar-Zvi, 2011). Here, our data showed that exogenous expression of *BnABI3* affects auxin-induced LR development, showing that *BnABI3* may facilitate ABA and auxin interaction. The reduced response of *abi3* mutants to IAA and NPA in LR initiation indicates that the presence of ABI3 is required for precise auxin signaling perception in the roots. Taken together, it is likely that the BnABI3 protein plays important roles in integrating developmental signals and external cues for plant growth.

## Author Contributions

WC and PX conceived and supervised the study, analyzed the data, and wrote the manuscript. PX conducted the experiments. All authors read and approved the manuscript.

## Conflict of Interest Statement

The authors declare that the research was conducted in the absence of any commercial or financial relationships that could be construed as a potential conflict of interest.

## Abbreviations

ABA: abscisic acid
ABI3: ABSCISIC ACID INSENSITIVE3
ER: endoplasmic reticulum
FLC: FLOWERING LOCUS C
FLM: FLOWERING LOCUS M
IAA: indole acidic acid
LR: lateral root
NPA: *N*-1-naphthylphthalamic acid
SRG: stay-green gene
TM: tunicamycin
