# Supplementary material for: Function of Brassica napus BnABI3 in Arabidopsis gs1, an Allele of AtABI3, in Seed Development and Stress Response
Source: Front Plant Sci. 2019 Feb 5;10:67. doi: 10.3389/fpls.2019.00067 (PMC6370748; doi:10.3389/fpls.2019.00067)
Supplement: Supplementary file 1 [file Data_Sheet_1.docx]

## Supplementary data

Article title: Function of *Brassica napus BnABI3* in *Arabidopsis gs1,* an allele of *AtABI3,* in seed development and stress response

Peipei Xu^1^, Weiming Cai^1*^

^1^Laboratory of Photosynthesis and Environment, CAS Centre for Excellence in Molecular Plant Sciences, Shanghai Institute of Plant Physiology and Ecology, Chinese Academy of Sciences, No. 300 Fenglin Road, Shanghai 200032, China

**Figure S1. Characterization of constitutive overexpression *BnABI3* transgenic *Arabidopsis*.**

**
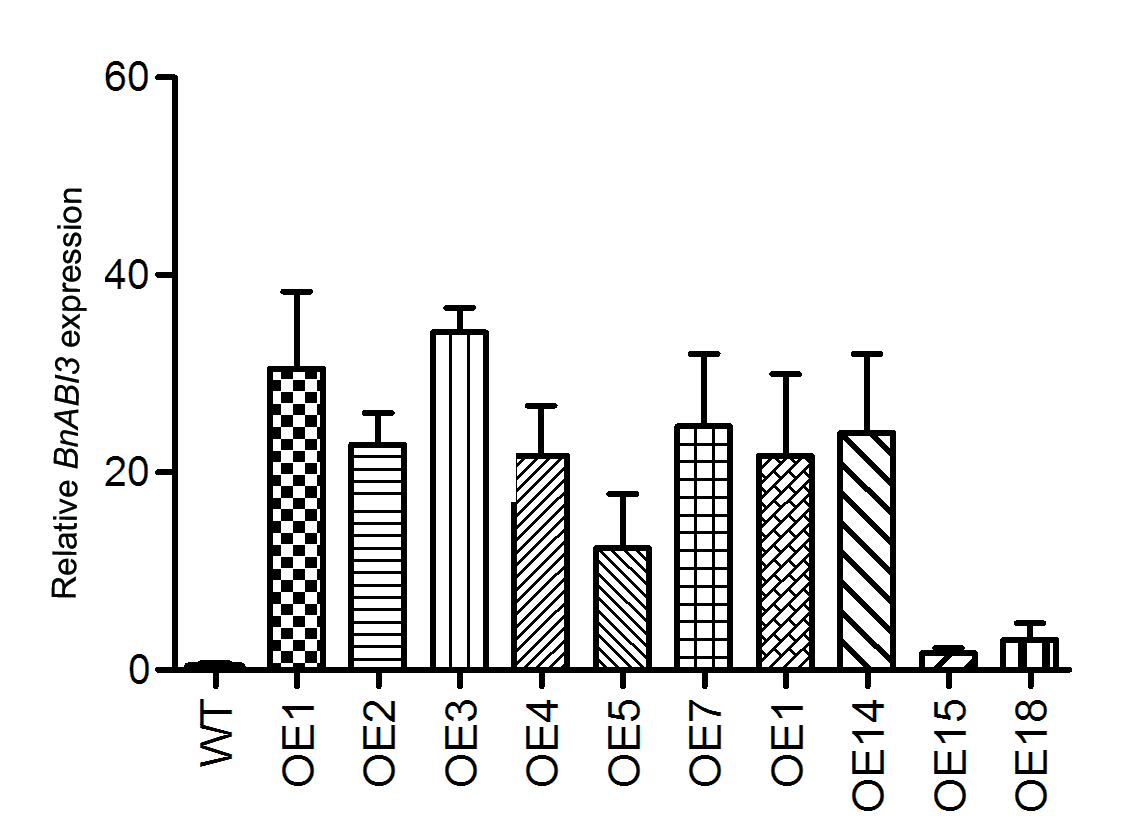
**

*BnABI3* expression levels in the 35S::*BnABI3* transgenic *Arabidopsis*. Values are given as mean ± SD, n=4.

**Figure S2.** Check the expression of truncated form of *AtABI3* at mRNA level in *bs1* mutant.


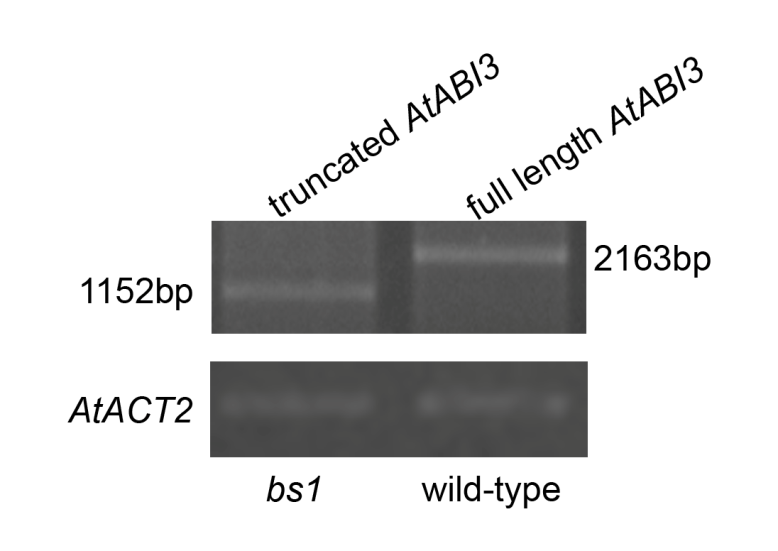


RT-PCR analysis of 1155bp truncated *AtABI3* gene expression in *bs1* mutant background and full length *AtABI3* gene expression in wild-type background.

**Figure S3. The *BnABI3* gene expression level in the wild-type and the *gs1* mutant complementary transgenic plants (*gs1*&proAtABI3::*BnABI3*).**


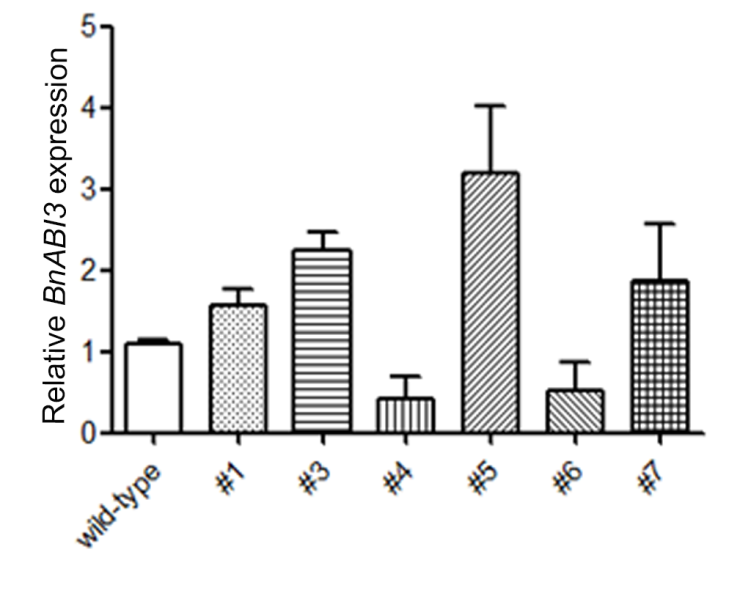


The *BnABI3* RT-qPCR primer is located at the conserved region between *AtABI3* and *BnABI3.* Values are given as mean ± SD, n=3.

**Figure S4**. **Alignment of the deduced amino acid sequences of AtABI3 and BnABI3. Identical amino acids among all aligned sequences are indicated with a star. The analysis was based on the deduced amino sequences of BnABI3 (LOC103860248) and AtABI3 (AT3G24650).**


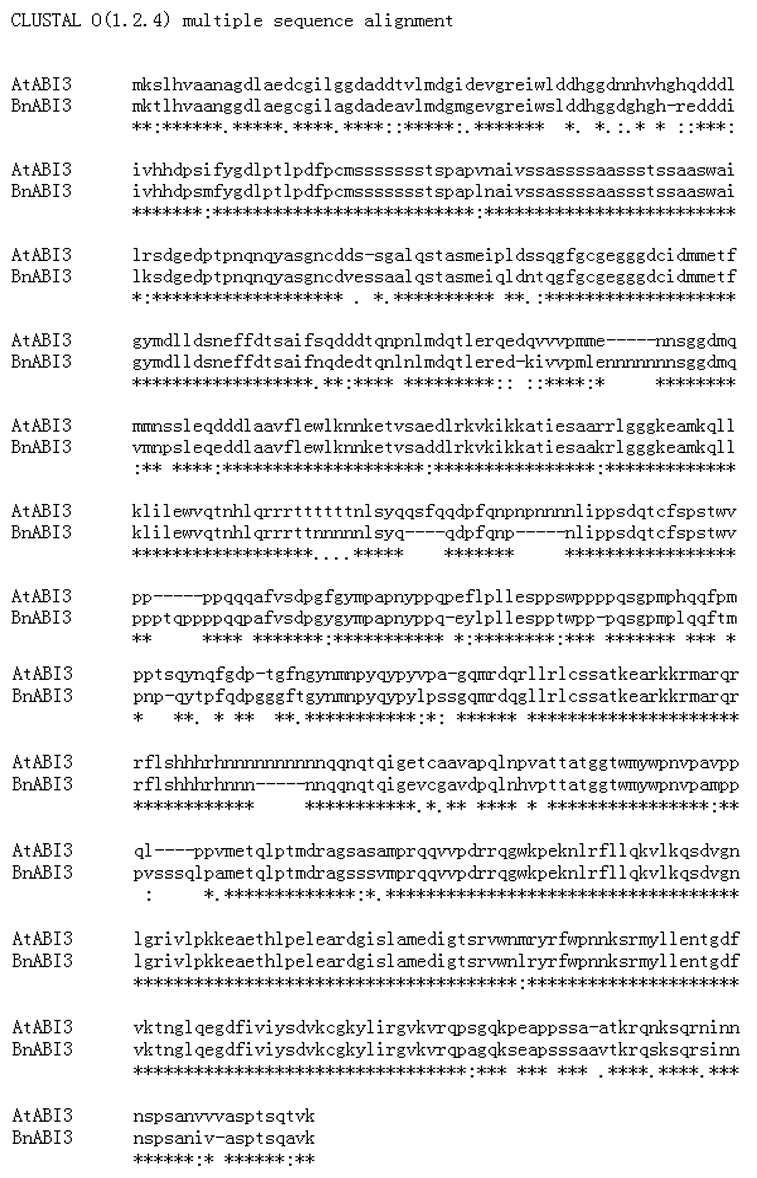


**Figure S5**. *SRG1* and *SRG2* genes expression levels during cold acclimation in wild type and *BnABI3* plants rosette leaves.


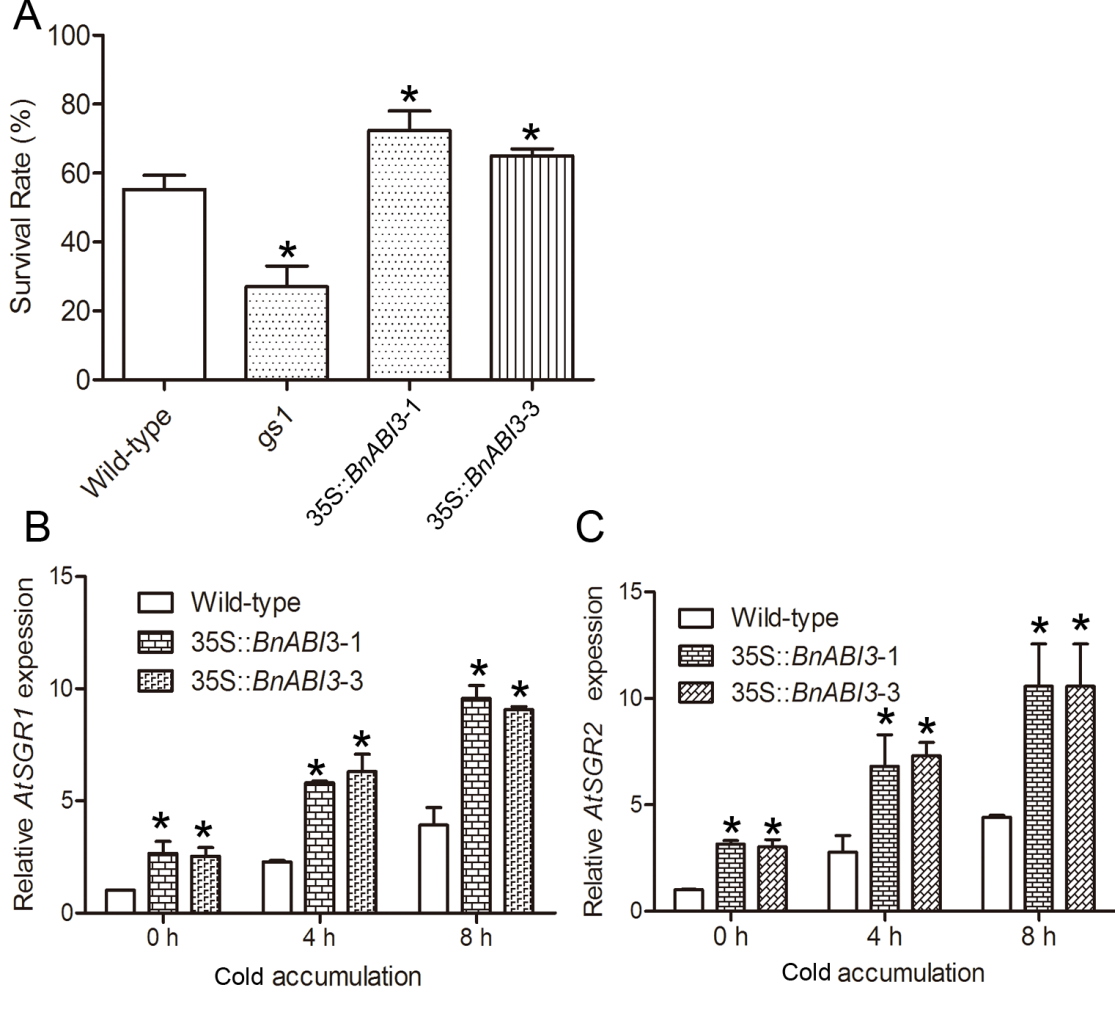


**(A)** Survival rate analysis of wild-type (Col-0), *gs1* and 35S::*BnABI3* transgenic plants after -8 °C freezing treatment. Expressions levels of **(B)** *AtSGR1* and **(C)** *AtSGR2* during 4°C cold accumulation for 0 h, 4 h and 8 h. Values are given as mean ± SD, n=4. **p*< 0.05 Student *t* test.

**Figure S6.**Inducible overexpression *BnABI3* by DEX treatment down-regulate TM stress tolerance.
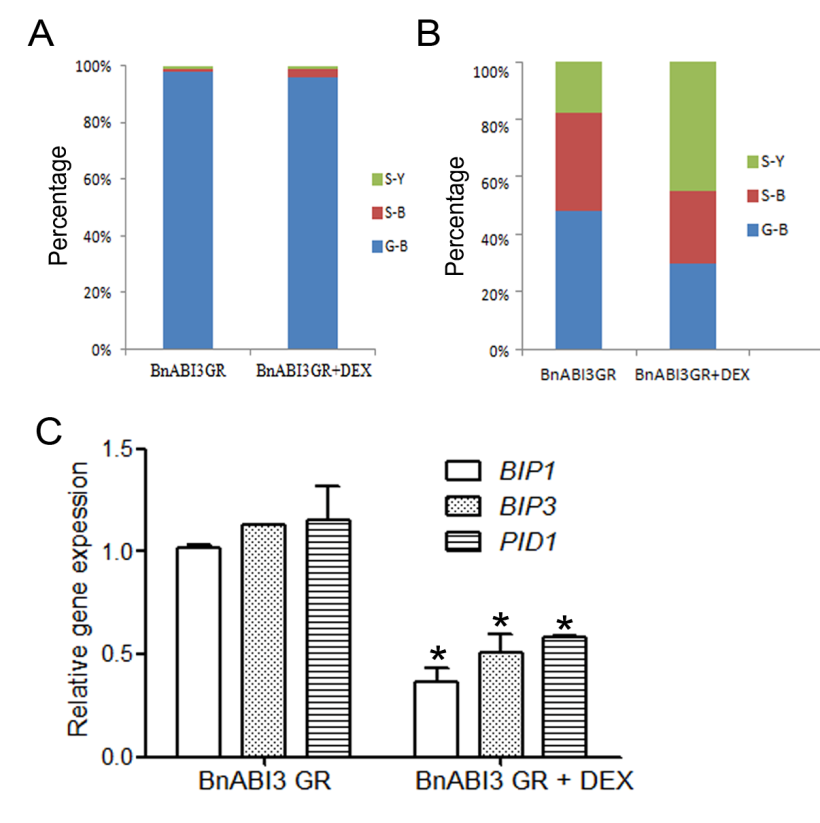


**(A, B)** The percentage of Green-Big (G-B), Green-Small (G-S) and Yellow-Small (Y-S) plants were calculated. The difference between the 7-day-old BnABI3GR transgenic *Arabidopsis* seedlings grown on MS medium supplied without or with DEX + 80 ng/ml tunicamycin (TM) treatments. **(C)** RT-qPCR analysis of *BIP1*, *BIP3* and *PID1* expression levels before and after treatments. A group of ten plants was measured for each genotype. Values are given as mean +SD, n=3.**p*<0.05 by Mann and Whitney test.

**Table S1 Primers used in plasmid constructions**

| **Transgenes** | **Primers (Sequence 5’-3’)** |
| --- | --- |
| Clone *BnABI3*cDNA | 5'-TCAGATCTACAAGCAAAAGT-3′^a^ |
|  | 5'-AAAACATCTCGAGAAAAAAG-3′^a^ |
| Pro-*AtABI3* | 5'-CTGCAG TTAAATGTTTGTAAGTATTATGA-3′^b^ |
|  | 5'-GGGCCCCGTTGAAGTGGAAATGAAACA-3′^b^ |
| double 35S:*BnABI3* | 5'-GGATCCATGAAAACCTTGCATGTGGCGG -3′^c^ |
|  | 5'-CTAGA**A**TCATTTAACTGCTTGAGAAGTTG -3′^c^ |
| Pro-*AtMUM1* | 5'-CTGCAG AGATAGGCTTATACATTATACA-3′ |
|  | 5'-GGGCCCAAGATCGAGCTGCTATAAAAGG-3′ |
| Pro-*AtGATL5* | 5'-CTGCAG CTAACCATGCATTTTTACAAAA-3′ |
|  | 5'-GGGCCCTGCGAGGAATGCAGAAGAAACT-3′ |
| Pro-*AtSRG1* | 5'-CTGCAG ACCGTCACGGAGATGAAGATG-3′ |
|  | 5'-GGGCCCCTGCTCTCTTGAAACCCAAATC-3′ |
| Pro-*AtSRG2* | 5'-CTGCAG CTGGCTTTTAATTATTAGTTT-3′ |
|  | 5'-GGGCCCTTTGCTTGTTCTCAAAAATAT-3′ |
| Pro-*AtPMEI6* | 5'-CTGCAG GTGTTCGATGACTCTGACATGC -3′ |
|  | 5'-GGGCCCTCTTTATTGGAGGAATCCAAAC-3′ |

Underlined are restriction digestion sites.

a: primers designed according to 5’/3’-UTR used for clone*BnABI3* gene cDNA in *Brassica napus*.

b: primers for genomic DNA for complementary assay.

c: primers for constitutive overexpression *BnABI3* gene.

**Table S2 Gene-specific primers used in RT-qPCR experiments**

| **Genes** | **Primers (Sequence 5’-3’)** |
| --- | --- |
| *ACTIN2* | 5’-TCAGATGCCCAGAAGTCTTGTTC-3’ |
|  | 5’-CCGTACAGATCCTTCCTGATATC-3’ |
| *UBQ10* | 5’- CGCCTCTAATCT CGCAGTTCC -3’ |
|  | 5’- GTTGTCAATGGTG TCGGAGGA-3’ |
| *AtABI3* | 5'-GACCAGGTCGTTGTTCCGAT-3′ |
|  | 5'-AATCCTCAGCCGACACAGTC-3′ |
| *BnABI3* | 5'-TACCAACACTCCCTGACTTCCCA-3′ |
|  | 5'-AGTTGAGGAAGAAGCAGCGGAA-3′ |
| *AtSGR1* | 5'-AGCAGCAGCAGCTCACTCTTCTT-3' |
|  | 5'-CCTAGGGAGCGTTGAAGGAT-3' |
| *AtSGR2* | 5'-ATTAGCGGAGGCCACTTCTT-3' |
|  | 5'-GTTGTACTCCGGGATGTTGG-3' |
| *AtFLC* | 5'-CTTGTGGATAGCAAGCTTGTG-3' |
|  | 5'-CATGAGTTCGGTCTTCTTGGC-3' |
| *AtFT* | 5'-TACGAAAATCCAAGTCCCAC-3' |
|  | 5'-AAACTCGCGAGTGTTGAAGT-3' |
| *AtSOC1* | 5'-CGAGCAAGAAAGACTCAAG-3' |
|  | 5'-TTCATGAGATCCCCACTTTT-3' |
| *AtFLM* | 5'-CTTGAGACTGCTCTGTCCGT-3' |
|  | 5'-CCAGAACCTGGTTCTCTTCT-3' |
| *AtPDI1* | 5'-GTTTCATCACGGCGATCACG-3' |
|  | 5'-CGAAGCTATTGTTGCCGACG-3' |
| *AtBIP1* | 5'-CCACTCACCAGAGCTCGTTT-3' |
|  | 5'-CCATGGCCTTCTTCACAGGT-3' |
| *AtBIP3* | 5'-GTGATCACGGTTCCAGCGTA-3' |
|  | 5'-TACGAACCACGTTAAGCCCC-3' |
| *AtAP1* | 5'-GACTTGCAAGCAATGAGCCC-3' |
|  | 5'-GCATGCTGTTTTGCTCCTGT-3' |
| *AtGATL5* | 5'-TCAAACCCCAACAAAACTCCGA-3' |
|  | 5'-GATCGAATTGACGGCTGCGATT-3' |
| *AtMUM1* | 5'-AGAGTAATTGGGAAGCTGACA-3' |
|  | 5'-CAACTGGATCAGGAGAAACCT-3' |
| *AtPMEI6* | 5'-CTTCTTCTTCTCCTATCACGTTT-3' |
|  | 5'-TCTAACGTATGTGCTGTACTGC-3' |

**Table S3 Gene-specific primers used in Chip-qPCR experiments**

| **Genes** | **Primers (Sequence 5’-3’)** |
| --- | --- |
| *ACTIN2* | 5’-CAGATGCCCAGAAGTCTTGTT-3’ |
|  | 5’-CGTACAGATCCTTCCTGATAT-3’ |
| *AtGATL5*-D1 | 5’-AACCATGCATTTTTACAAAA-3’ |
|  | 5’-TTTATCGATTACTTTCCAGCA-3’ |
| *AtMUM1*-D2 | 5'-TTTCCAAACTAACAAATAAA-3′ |
|  | 5'-TAAATAAAAGATTACATTCA-3′ |
| *AtMUM1*-D3 | 5'-AATGAATATCCTATGCATTTT-3′ |
|  | 5'-ATGAAACTAATTCAAATTCTA-3′ |
| *AtSGR1*-D1 | 5'-CCCACCATGACGACACATGG-3' |
|  | 5'-TGTTGCTTCACGTTTCACAC-3' |
| *AtSGR2*-D2 | 5'-GAACTATAACCGCAATTAAAG-3' |
|  | 5'-AGTTCTTCAAAGATCTCTTTGA-3' |
